# Sleep Deprivation Aggravates Periodontitis Through Trigeminal‐Periodontal Neuroimmune Pathway Mediated by the AChE‐ACh‐α7nAChR Axis

**DOI:** 10.1002/advs.202500945

**Published:** 2025-08-14

**Authors:** Kehao Liu, Qi Huang, Mingcong Yang, Ziyu Huang, Kamoran Tuerhong, Yue Zu, Ying Xie, Xuehui Hu, Qianyu Zhang, Ping He, Nannan Huang, Rong Zhang, Yuzhou Li, Sheng Yang

**Affiliations:** ^1^ College of Stomatology Chongqing Medical University Chongqing 401147 P. R. China; ^2^ Neuroscience Research Institute Peking University Beijing 100871 P. R. China; ^3^ Department of Neurobiology Department of Integration of Chinese and Western Medicine School of Basic Medical Sciences Peking University Beijing 100871 P. R. China; ^4^ Key Laboratory for Neuroscience Ministry of Education Key Laboratory for Neuroscience Ministry of Health Beijing 100871 P. R. China; ^5^ Autism Research Center of Peking University Health Science Center Beijing 100871 P. R. China; ^6^ Department of Integration of Chinese and Western Medicine School of Basic Medical Sciences Peking University Beijing 100871 P. R. China; ^7^ Chongqing Municipal Key Laboratory of Oral Biomedical Engineering of Higher Education Chongqing Key Laboratory of Oral Diseases and Biomedical Sciences Chongqing 401147 P. R. China

**Keywords:** α7nAChR, cholinergic anti‐inflammatory pathway, electroacupuncture, macrophage, neuroimmune, periodontitis, sleep deprivation

## Abstract

Continuous sleep deprivation (SD) triggers systemic inflammatory storm and immune dysregulation, yet its specific impact on periodontitis and the corresponding therapeutic interventions remains unclear. Consequently, this study elucidates the neuroimmune mechanisms linking SD to ligature‐induced periodontitis (LIP) in mice and evaluates electroacupuncture (EA) as a novel adjunctive therapy. Screening analyses (ELISA, public databases, flow cytometry, immunofluorescence, etc.) identified pivotal roles of acetylcholine (ACh), α7 nicotinic acetylcholine receptor (α7nAChR), and acetylcholinesterase (AChE) in SD‐aggravated periodontitis with a decrease in ACh levels, down‐regulation of α7nAChR on macrophages, and an increase in trigeminal ganglion‐derived AChE. Clinical validation in periodontitis patients with poor sleep (PSQI ≥ 5) confirmed this tripartite cholinergic imbalance. Ultimately, both in vivo and in vitro data demonstrated that EA inhibits M1 polarization while promoting M2 polarization of macrophages through α7nAChR activation. Therefore, SD exacerbates periodontitis via the AChE‐ACh‐α7nAChR axis‐mediated trigeminal‐periodontal neuroimmune pathway, whereas EA partially reverses this pathology by targeting macrophage α7nAChR. These findings reveal new insights into the “oral‐brain axis” in oral disease pathogenesis and provide novel therapeutic strategies for periodontitis patients with comorbid sleep disorders.

## Graphical abstract

Sleep deprivation (SD) exacerbates ligature‐induced periodontitis (LIP) through the trigeminal nerve‐periodontal neuroimmune pathway mediated by the acetylcholinesterase (AChE)‐acetylcholine (ACh)‐α7 nicotinic receptor (α7nAChR) axis. While electroacupuncture (EA) alleviates the condition of LIP combined with SD by activating the α7nAChR.

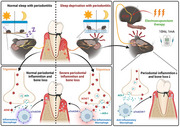

## 1 Introduction

Sleep disorders, arising from dysregulation of sleep centers, encompass a broad spectrum of systemic repercussions, ultimately influencing the functionality and condition of peripheral organs via neurological, vascular, and endocrine disruptions.^[^ ^1^, ^2^, ^3^ ^]^ Emerging evidence suggests an association between certain sleep disorders—particularly insomnia, sleep deprivation, and shift work‐related disturbances—and increased population‐level risk of periodontitis.^[^ ^4^, ^5^, ^6^ ^]^ However, a direct causal or aggravating role of sleep disorders in periodontitis pathogenesis remains unconfirmed due to limited mechanistic studies.

Neuroimmune pathways are implicated in sleep‐related inflammatory disorders.^[^ ^7^ ^]^ Nicotinic acetylcholine receptors (nAChRs) constitute one of the most prevalent receptor subtypes within the central nervous system (CNS) and serve as a crucial element of the cholinergic anti‐inflammatory pathway (CAP), whose activation regulates the expression of inflammatory factors and exerts an immunomodulatory effect on local and systemic inflammation.^[^ ^8^ ^]^ In CAP, Acetylcholine—synthesized in neurons, transported axonally, stored in vesicles, and released synaptically—binds nAChRs on target cells to exert anti‐inflammatory effects.^[^ ^9^ ^]^ Significantly, α7nAChR is highly expressed at mucosal barriers (e.g., gut, periodontium), suggesting therapeutic potential in periodontitis.^[^ ^10^, ^11^ ^]^ Intriguingly, experimental sleep deprivation reduces α7nAChR expression in rodent brain microglia, impairing cognition.^[^ ^12^ ^]^ Critically, whether sleep deprivation similarly disrupts periodontal cholinergic signaling in inflammatory contexts is unknown.

It is well‐documented that sleep disorders represent inflammatory states that disrupt the overall immune system of the body, primarily via the activation of pro‐inflammatory cells like neutrophils, monocytes, and T lymphocytes, as well as the upregulation of C‐reactive proteins and other inflammatory mediators.^[^ ^13^ ^]^ While periodontitis is often viewed as a localized immunopathy, host immune responses critically influence both systemic and local inflammatory progression.^[^ ^14^, ^15^ ^]^ This underscores the need for adjunctive periodontal therapies targeting systemic‐local immune homeostasis.

Electroacupuncture (EA) is a minimally invasive therapeutic approach that integrates physical therapy with the theoretical and technical principles of traditional Chinese acupuncture. EA stimulates specific areas of the body, known as “acupuncture points” in traditional Chinese medicine, to regulate distant physiological functions and treat various diseases.^[^ ^16^ ^]^ Extensive validation has firmly established EA as a highly effective treatment for sleep disorders and periodontitis in clinical trials and animal experiments, respectively.^[^ ^17^, ^18^, ^19^, ^20^ ^]^ The anti‐inflammatory properties of EA are intricately linked to the CAP. For instance, Yang et al. reported that EA activates the α7nAChR‐mediated JAK2/STAT3 signaling pathway in macrophages, thereby significantly improving intestinal inflammation.^[^ ^21^ ^]^ Similarly, Ding et al. further demonstrated that EA alleviates inflammation in dry eye syndrome by modulating the α7nAChR/NF‐κB signaling pathway.^[^ ^22^ ^]^ However, EA's mechanism in comorbid sleep disorder‐periodontitis remains unelucidated.

In this study, we combined experimental sleep deprivation (SD) with ligature‐induced periodontitis (LIP) to investigate SD's impact on periodontal inflammation. Following this, EA was administered at the distal Zusanli acupoint. Given the cholinergic system's dual relevance to SD pathology and EA therapy, we hypothesized that SD exacerbates periodontitis—while EA mitigates it—through neuroimmune modulation of cholinergic pathways.

## 2 Results

### 2.1 SD Exacerbate LIP and the Mitigating Effects of EA

**Figure 1:**
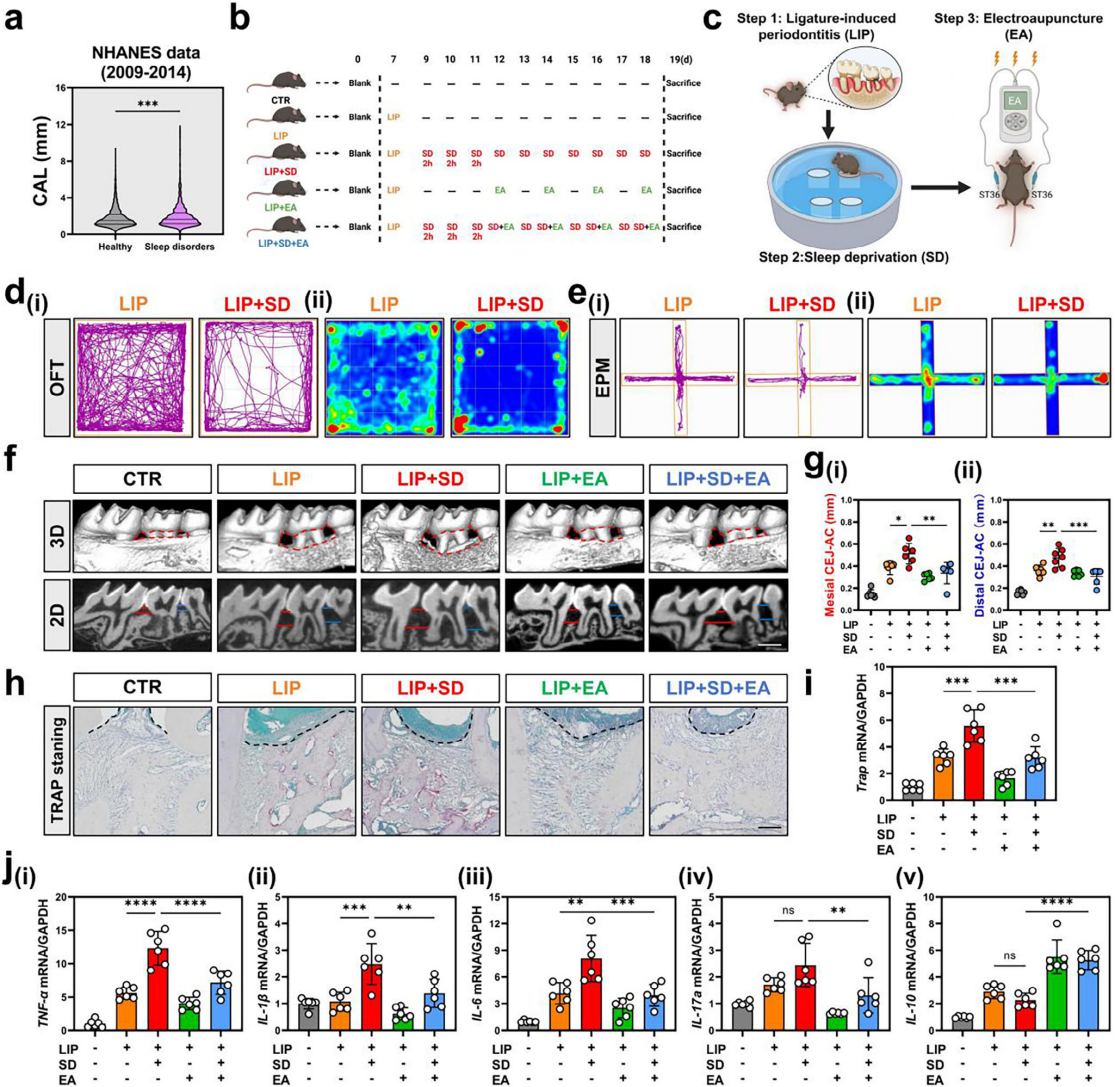
Sleep deprivation (SD) exacerbates alveolar bone resorption and inflammatory response in ligature‐induced periodontitis (LIP) mice, and electroacupuncture (EA) stimulation delays this process. a) Bar chart of clinical attachment loss (CAL) in the healthy control group versus the sleep disorder group from the NHANES database between 2009 and 2014. b) Timeline of an experiment into LIP combined SD and EA treatment. c) Schematic diagram of LIP + SD and EA treatment in mice. d,e) Representative trajectories i) and heat maps ii) in OFT and EPM. f) 3D reconstruction of micro‐CT scanned images of the alveolar bone. Scale bar = 1 mm. (g) Comparison of the mesial i) and distal ii) CEJ–ABC distances of alveolar bone loss. (h) Histological evaluation of osteoclasts by tartrate‐resistant acid phosphatase (TRAP) staining. Scale bar = 100 µm. i) Periodontal tissues mRNA expression of osteoclast‐related genes TRAP. (j) Periodontal tissues mRNA expression of pro‐inflammatory‐related genes TNF‐α (i), IL‐1β (ii), IL‐6 (iii), IL‐17a (iv), and anti‐inflammatory‐related gene IL‐10 (v). Data are shown as the mean ± standard error of the mean (SEM) (n = 6 per group). ^*^ *p* <0.05; *^*^ *p* <0.01; **^*^ *p* <0.001; ***^*^ *p* <0.0001.

By integrating the statistics of the cohort in the NHANES database who recorded both sleep and periodontal health data from 2009 to 2014, we found that individuals with sleep disorders exhibited higher periodontal clinical attachment loss compared to the healthy cohort, while no significant difference was observed in probing depth (**Figure** **1**a; Figure S1a, Supporting Information). This suggests that the exacerbation of periodontitis may be associated with sleep disorders. To explore the impact of sleep disorders on periodontitis and assess the therapeutic efficacy of EA, we successfully established a mouse model of ligature‐induced periodontitis combined with sleep deprivation (LIP + SD) through comprehensive evaluations encompassing weight analysis, serum corticosterone levels, and behavioral assessments (open‐field test and elevated plus maze) (Figure 1d,e; Figure S1b–g, Supporting Information). Subsequently, EA was introduced as a potential therapeutic intervention in this model (Figure 1b,c). 3D reconstructions and sagittal micro‐CT images of the maxillary molar region revealed that SD exacerbated alveolar bone resorption in mice with LIP, while EA effectively alleviated the SD‐exacerbated resorption (Figure 1f). Quantitative measurements demonstrated severe bone loss in the LIP + SD group compared to the LIP‐only group, both mesially (0.513 mm versus 0.385 mm, *p *<0.05) and distally (0.554 mm vs 0.361 mm, *p *<0.05) (Figure 1g). Notably, EA significantly reduced the bone resorption in both LIP‐only group (mesially: 0.305 mm, *p *<0.05; distally: 0.337 mm, *p *<0.05) and LIP + SD group (mesially: 0.337 mm, *p *<0.05; distally: 0.307 mm, *p *<0.05) (Figure 1f,g). Additionally, our findings also indicated that SD alone did not induce periodontitis (Figure S1h,i, Supporting Information), suggesting that SD operates as a systemic factor that exacerbates pre‐existing periodontitis.

The micro‐CT results above suggested the activation of osteoclasts within the periodontal tissue. To further corroborate this hypothesis, TRAP staining was conducted. Compared to the LIP group, the LIP + SD group exhibited a scattered accumulation of periodontal osteoclasts in the alveolar bone (Figure 1h). Notably, after EA treatment, irrespective of SD administration, a marked reduction in osteoclasts was observed around the second molars (Figure 1h). Subsequently, we analyzed TRAP expression at the transcriptional level across all groups, revealing a trend that aligned with the TRAP staining findings (Figure 1i).

Periodontal tissues were collected for qPCR to quantify the levels of inflammatory markers in each group. The results revealed that the mRNA levels of pro‐inflammatory cytokines, namely TNF‐α, IL‐1β, and IL‐6, but not IL‐17a, were elevated in the LIP + SD group compared to the LIP group (Figure 1j). This finding implies that, under these conditions, the innate immune system may play a more prominent role than Th17 cells, which are adaptive immune cells known to contribute to periodontal disease progression.^[^ ^23^ ^]^ Following EA treatment, there was a notable decrease in the levels of these pro‐inflammatory cytokines, highlighting the broad inhibitory effect of EA on cytokine production (Figure 1i). Regarding the anti‐inflammatory cytokine, the mRNA expression of IL‐10 in the LIP + SD group was comparable to that in the LIP group. However, EA significantly up‐regulated the level of IL‐10 (Figure 1j). Collectively, these results suggest that SD exacerbates LIP by promoting alveolar bone resorption, activating osteoclasts, and promoting inflammatory responses, while EA can partially reverse this trend.

### 2.2 Localized Dysregulation of α7nAChR Expression in Periodontal Tissue Under SD

**Figure 2:**
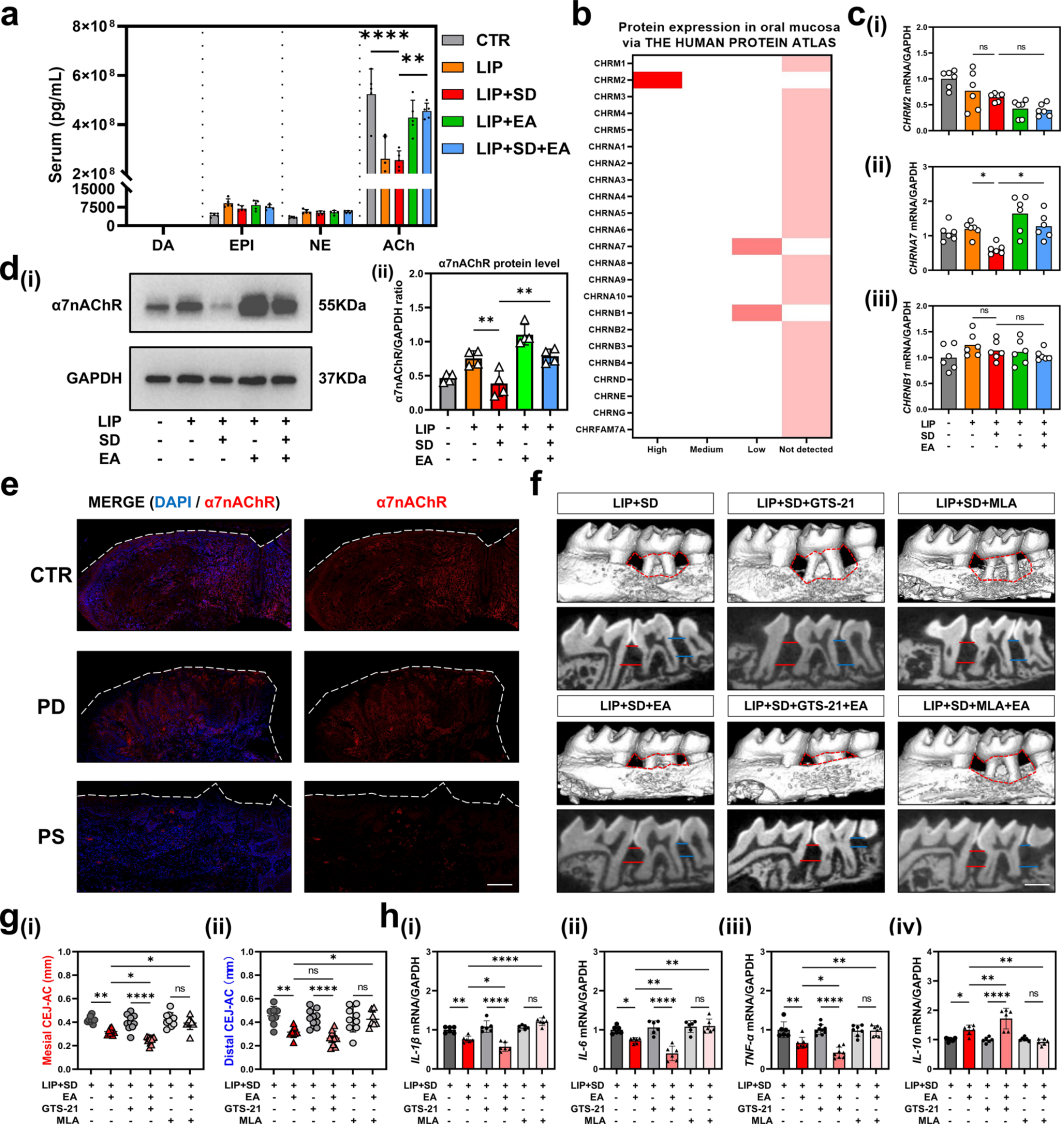
The effects of SD and EA are mediated by inhibition/activation of α7nAChR in the cholinergic pathway. a) Mouse serum levels of neurotransmitters dopamine (DA), epinephrine (EPI), noradrenaline (NE), and acetylcholine (ACh) at the end of the experiment (10:00 AM on the 19th day). b) Heat map of ACh receptors expression in human oral mucosa via THE HUMAN PROTEIN ATLAS database. c) Mouse periodontal tissues mRNA expression of CHRM2 (i), CHRNA7 (ii), and CHRNB1 (iii). d) Western blots of α7nAChR in mouse periodontal tissue. (e) Immunofluorescence staining of human gingival tissue with DAPI and α7nAChR. Scale Bar: 100 µm. f) 3D reconstruction of micro‐CT scanned images of the mouse alveolar bone. Scale bar = 1 mm. g) Comparison of the mesial (i) and distal (ii) CEJ–ABC distances of mouse alveolar bone loss. h) Mouse periodontal tissues mRNA expression of pro‐inflammatory‐related genes IL‐1β (i), IL‐6 (ii), TNF‐α (v), and anti‐inflammatory‐related gene IL‐10 (iv). Data are shown as the mean ± standard error of the mean (SEM) (n = 6 per group). ^*^ *p* <0.05; ^**^ *p* <0.01; ^***^ *p* <0.001; ^****^ *p* <0.0001.

To delve deeper into how SD exacerbates periodontitis and the therapeutic mechanism of EA, we conducted a screen for serum neurotransmitters that have been previously reported as significant.^[^ ^24^, ^25^ ^]^ The results indicated that ACh, a neurotransmitter of the cholinergic pathway, was notably decreased in both the LIP and LIP + SD groups, but increased in the EA group. In contrast, neurotransmitters of the catecholamine pathway, such as dopamine, epinephrine, and norepinephrine, showed no significant changes (**Figure** **2**a), thereby implying a specific role of CAP in mediating the effects of electroacupuncture therapy. Furthermore, to establish a connection between systemic neurotransmitters and local periodontal inflammation, we analyzed data from the HUMAN PROTEIN ATLAS and discovered that only three receptors for ACh (*CHRM2*, *CHRNA7*, and *CHRNB1*) are expressed in periodontal tissue (Figure 2b). These receptors were further validated through qPCR, revealing that only CHRNA7 expression was decreased in the LIP + SD group compared to the LIP group, and increased in the EA group (Figure 2c). Similarly, analysis of α7nAChR at the protein level showed trends consistent with the qPCR results (Figure 2d). Furthermore, the expression of α7nAChR was significantly inhibited in the human gingival tissue of the PS group [periodontitis with poor sleep quality (PSQI ≥ 5)] (Figure 2e), indicating that α7nAChR may plays a role in exacerbating PD under SD. These results collectively suggested that SD is associated with the down‐regulation of α7nAChR and EA up‐regulated the α7nAChR, highlighting the importance of CAP in sleep‐deprived periodontitis.

To further elucidate the role of α7nAChR, we applied pharmacological agents, specifically the α7nAChR agonist GTS‐21 and the α7nAChR inhibitor MLA, to the SD + LIP and SD + LIP + EA groups. Micro‐CT revealed that, in the LIP + SD group, the use of GTS‐21 or MLA alone, without EA treatment, did not significantly alter alveolar bone resorption. Notably, when GTS‐21 or MLA were administered in combination with EA in the LIP + SD + EA group, GTS‐21 was found to inhibit the progression of bone resorption, whereas MLA exacerbated it (Figure 2f,g). To gain insight into the underlying mechanisms, we examined inflammatory cytokines in the periodontal tissue. Compared to the LIP + SD + GTS‐21 group, the LIP + SD + EA + GTS‐21 group exhibited significantly reduced levels of proinflammatory cytokines (IL‐1β, IL‐6, TNF‐α) and upregulated IL‐10. In contrast, no difference was observed between the LIP + SD + MLA group and the LIP + SD + EA + MLA group (Figure 2h). Intriguingly, despite its known agonistic effect, GTS‐21 did not reverse alveolar bone loss. To clarify this, we analyzed the α7nAChR protein expression. Our findings showed no obvious change in α7nAChR expression before or after GTS‐21 application (Figure S2, Supporting Information). Therefore, we speculated that, due to the low receptor expression in the periodontal tissue, GTS‐21 could not fully activate α7nAChR. However, EA seemed to up‐regulate the protein expression of α7nAChR (Figure 2d). Collectively, SD and EA act through inhibition/activation of α7nAChR mediated by the CAP.

### 2.3 SD Exacerbate LIP via Downregulation of α7nAChR in Periodontal Macrophages and Enhancement of AChE in the Trigeminal Ganglion

**Figure 3:**
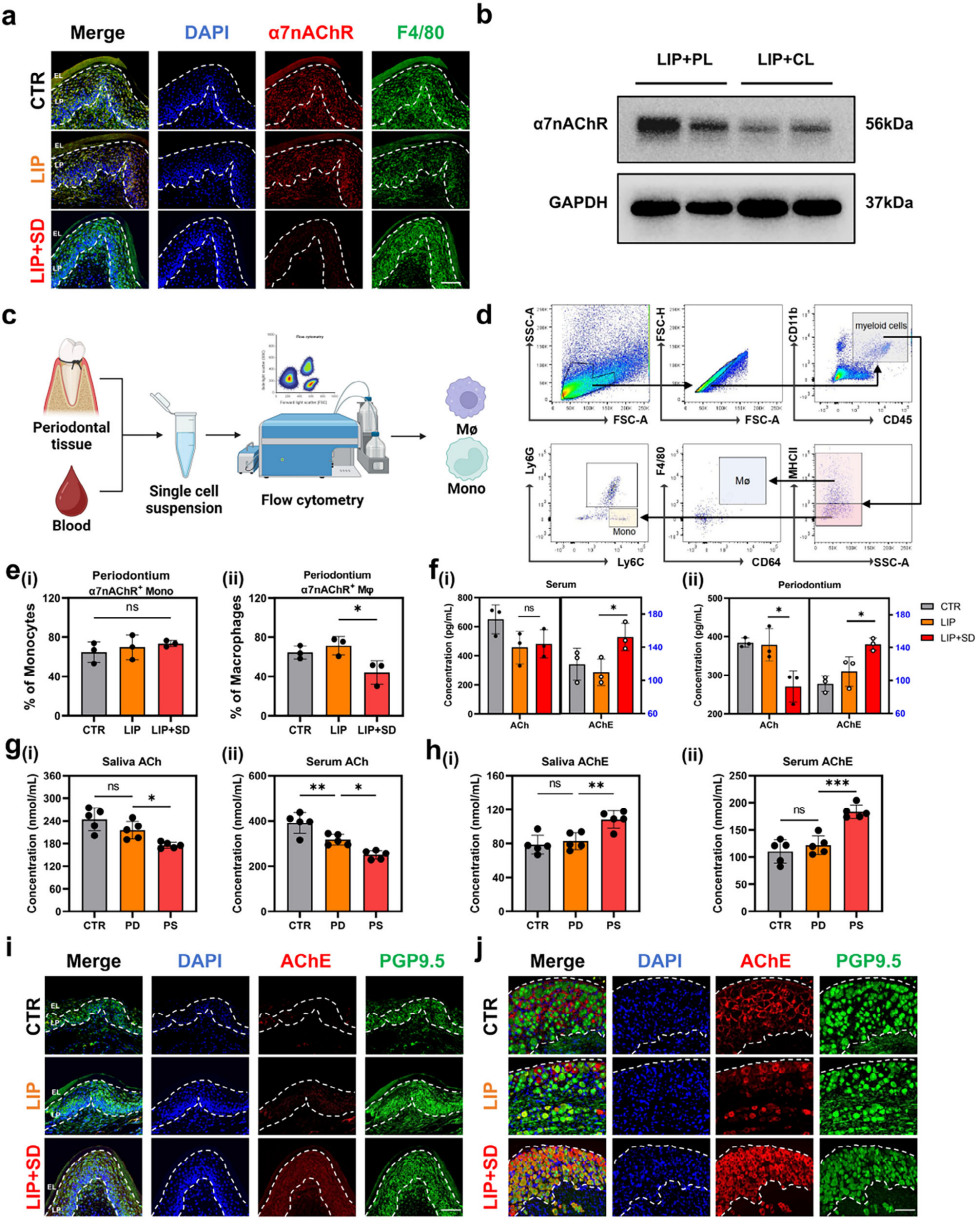
SD acts by down‐regulating α7nAChR on periodontal macrophages and enhancing AChE in the trigeminal ganglion. a) Co‐expression of α7nAChR and macrophage marker F4/80 in mouse periodontal tissue. Scale Bar: 50 µm. EL: epithelium; LP:Lamina Propria. b) Western blots of α7nAChR in mouse periodontal tissue. c) Schematic diagram of periodontal tissue and blood single‐cell suspension preparation for use in flow cytometry. d) Flow cytometric gating strategy of myeloid cells including macrophages (MΦ, CD45^+^CD11b^+^MHCII^+^CD64^+^F4/80^+^) and monocytes (mono, CD45^+^CD11b^+^MHCII^+^ Ly6C^+^). e) The frequencies of α7nAChR^+^ monocytes (i) and α7nAChR^+^ macrophages in periodontal tissues. f) Mouse serum (i) and periodontium (ii) levels of ACh and AChE. g) Human saliva (i) and serum (ii) levels of ACh. h) Human saliva (i) and serum (ii) levels of AChE. i) Co‐expression of AChE and neural marker PGP9.5 in mouse periodontal tissue. Scale Bar: 50 µm. j) Co‐expression of AChE and neural marker PGP9.5 in mouse trigeminal ganglion. Scale Bar: 50 µm. ^*^ *p* <0.05.

To investigate the mechanisms underlying the exacerbation of LIP by SD through the CAP, we conducted a comprehensive analysis of immune cell alterations in periodontal tissues. By utilizing periodontal immunofluorescence staining, we observed that the down‐regulation of α7nAChR in the LIP + SD group showed a potential association with F4/80^+^ cells, specifically macrophages, but not with CD3^+^ T cells or CD19^+^ B cells (**Figure** **3**a; Figure S3a,b, Supporting Information). Then, we employed clodronate liposomes to successfully deplete macrophages in mice (Figure S3e,f, Supporting Information). Subsequent analysis of periodontal tissue revealed a significant decrease in α7nAChR level in the LIP + CL group following macrophage depletion (Figure 3b). Meanwhile, flow cytometry analysis of periodontal tissue and blood samples from mice in the LIP + SD group demonstrated a reduction in macrophages but not monocytes within the periodontal tissue (Figure 3c–e). Notably, neither macrophages nor monocytes exhibited changes in the blood (Figure S3c,d, Supporting Information). These findings collectively suggest a specific association between the expression of α7nAChR and macrophages in periodontal tissues.

Under the normal physiological conditions, the CAP maintains a dynamic equilibrium between ACh and its hydrolase, acetylcholinesterase (AChE).^[^ ^26^ ^]^ To investigate this balance in the context of SD and LIP, we examined the levels of ACh and AChE in both mouse serum and periodontal tissue. Our results indicated that, in serum, there was no significant change in ACh level between the LIP and LIP + SD groups. However, an increase in AChE level was observed in the LIP + SD group compared to the LIP group. In mouse periodontal tissue, we found a decline in ACh level in the LIP + SD group, accompanied by a significant increase in AChE level compared to the LIP group (Figure 3f). Crucially, we also observed decreased ACh and increased AChE levels in both human saliva and blood samples from the PS group compared to the PD group (Figure 3g,h). These findings suggest a potential correlation between the decreased expression of α7nAChR and ACh and the increased activity of AChE, indicating a disruption in the CAP under SD conditions.

To elucidate the mechanism underlying the elevation of AChE in the periodontium, we employed fluorescent staining of periodontal tissues for co‐localization analysis. Our findings revealed that AChE^+^ cells predominantly coincided with PGP9.5^+^ cells (a pan‐neuronal marker widely used to label peripheral nerve fibers and sensory neurons), but did not appear to be associated with CD31^+^ or CD90^+^ cells (Figure 3i; Figure S3g,h, Supporting Information). Hence, we speculate that the increase in AChE might be linked to the neural pathway.

Given that the periodontal region is innervated by the trigeminal nerve,^[^ ^27^ ^]^ we further investigated the trigeminal ganglia of mice. Our results showed that the number of AChE^+^ cells in the LIP + SD group was significantly higher than that in both the CTR and LIP groups, and these cells co‐localized with PGP9.5^+^ cells (Figure 3j). Additionally, the expression of AChE in the LIP + SD group was markedly elevated compared to the LIP group (Figure S3i, Supporting Information). Collectively, these findings suggest that SD may exacerbate periodontitis by down‐regulating α7nAChR on peripheral macrophages and up‐regulating AChE in the trigeminal ganglion. This indicates a potential neural mechanism involved in the modulation of LIP by SD.

### 2.4 Trigeminal Ganglion‐Derived AChE Suppresses Periodontal Macrophage Cholinergic Signaling

**Figure 4:**
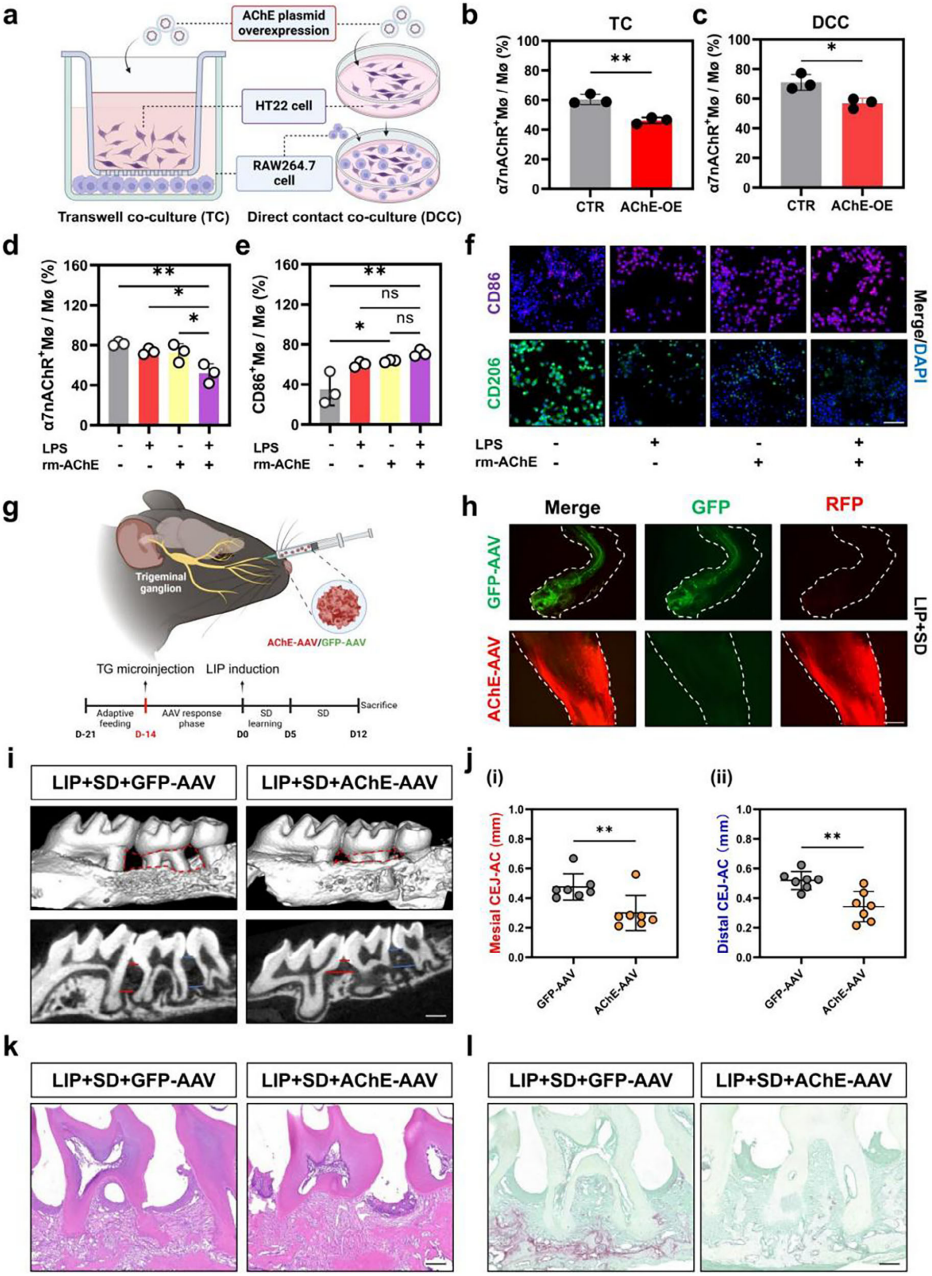
SD acts via AChE in the trigeminal pathway to influence the CAP on periodontal macrophages. a) Schematic diagram of co‐culture of HT22 cells and RAW264.7 cells in two different ways. b,c) The frequencies of α7nAChR^+^ macrophages in transwell co‐culture (b) and direct contact co‐culture (c). d,e) Frequency of α7nAChR^+^ macrophages (d) and CD86^+^ macrophages (e) after LPS‐stimulated intervention with AChE recombinant protein. f) Immunofluorescence stainings for CD86 and CD206 in RAW264.7 after AChE recombinant protein intervention in LPS environment. Scale Bar: 100 µm. g) Schematic and timeline diagram of microinjection of adeno‐associated virus into the trigeminal ganglion through the infraorbital foramen. h) Microscopic image of adeno‐associated virus in trigeminal ganglion. Scale Bar: 50 µm. i) 3D reconstruction of micro‐CT scanned images of the alveolar bone. Scale bar = 1 mm. j) Comparison of the mesial i) and distal (ii) CEJ–ABC distances of alveolar bone loss. k) Histological evaluation of Haematoxylin–eosin (H&E) staining. Scale bar = 200 µm. l) Histological evaluation of osteoclasts by TRAP staining. Scalebar = 200 µm. ^*^ *p* <0.05; ^**^ *p* <0.01.

To interrogate whether neuronal AChE affects α7nAChR on macrophages, we first overexpressed HT22 neuronal cells by using the AChE plasmid, and then co‐cultured HT22 neuronal cells and RAW264.7 macrophages using two co‐culture methods, the transwell and direct‐contact (**Figure** **4**a). Our research found that overexpressing AChE on HT22 cells results in down‐regulation of α7nAChR expression in RAW264.7, both in transwell co‐culture and direct contact co‐culture (Figure S4a, Supporting Information). Furthermore, flow cytometry results revealed that when overexpressing AChE on HT22, the proportion of α7nAChR^+^ RAW264.7 cells was reduced, both on two culture conditions (Figure 4b,c; Figure S4b,c, Supporting Information). We then treated RAW264.7 with AChE recombinant protein administered directly in an inflammatory environment and again found that the α7nAChR^+^ cell rate was significantly reduced (Figure 4d) and was able to promote an increase in the CD86^+^ cell rate (Figure 4e). Immunofluorescence staining also revealed a significant increase in CD86^+^ cells and decrease in CD206^+^ cells in RAW264.7 cells stimulated by LPS and AChE recombinant proteins, suggesting that AChE intervention has a tendency to promote macrophage M1 polarisation.

Subsequently, in LIP + SD mice we injected AChE adeno‐associated virus (AChE‐AAV) through the infraorbital foramen to ablate AChE in the trigeminal ganglion on one side, while leaving the contralateral side blanked. Additionally, we established a control group by injecting an AAV expressing green fluorescent protein (GFP‐AAV) (Figure 4g). Two weeks later, we collected trigeminal ganglia and observed their AAV‐mediated fluorescence by microscopy. We found that two AAVs were successfully injected into the trigeminal ganglion of LIP + SD mice (Figure 4h). Then by Micro‐CT and histological analyses, we found that the alveolar bone resorption and osteoclast activation in AChE‐AAV group was significantly lower than that of the GFP‐AAV group (Figure 4i–l); whereas the alveolar bone resorption on its contralateral untreated side was no different from that of the GFP‐AAV group (Figure S4d,e, Supporting Information). The above data suggest that SD exacerbates the local periodontal inflammation by promoting the transmission of AChE of trigeminal ganglion origin to the periodontium in order to inhibit the expression of the periodontal CAP.

### 2.5 EA‐Driven α7nAChR Activation Reverses Macrophage Pro‐Inflammatory Polarization

**Figure 5:**
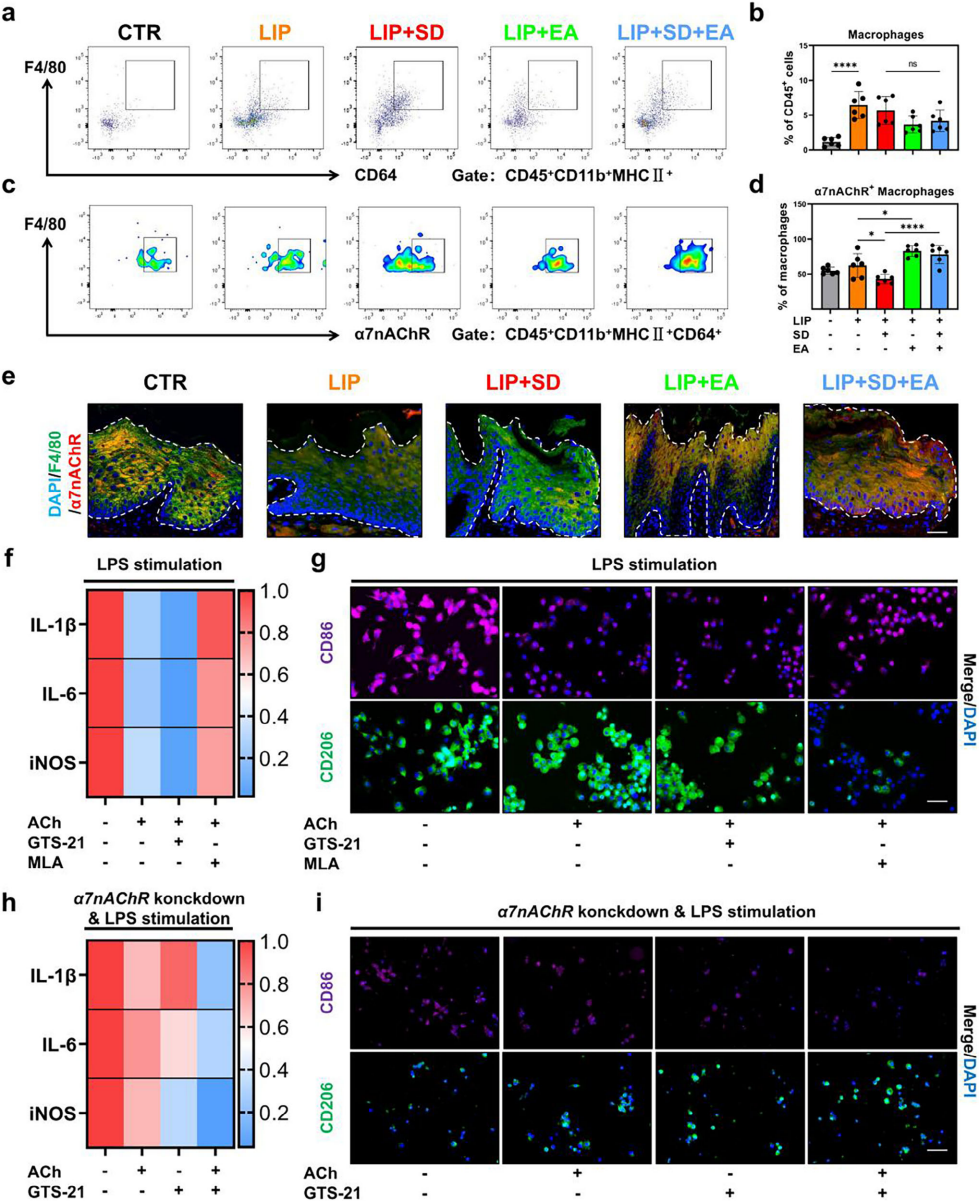
EA exerts anti‐inflammatory effects by activating α7nAChR on periodontal macrophages. a) Flow cytometric gate of periodontal macrophages (MΦ, CD45^+^CD11b^+^MHCII^+^CD64^+^F4/80^+^) in each group. Samples collected at Day 19. b) The frequencies of macrophages in periodontal tissues. c) Flow cytometric gating strategy of periodontal α7nAChR^+^ macrophages. d) The frequencies of α7nAChR^+^ macrophages in periodontal tissues. e) Co‐expression of α7nAChR and macrophage marker F4/80 in periodontal tissue. Scale Bar: 50 µm. mRNA expression of pro‐inflammatory‐related genes (IL‐1β, IL‐6, and iNOS) f) and immunofluorescence stainings for CD86 and CD206 g) in RAW264.7 after exogenous ACh supplementation and then activation/inhibition of α7nAChR in LPS environment. Scale Bar: 100 µm. mRNA expression of pro‐inflammatory‐related genes (IL‐1β, IL‐6, and iNOS) h) and immunofluorescence stainings for CD86 and CD206 i) in RAW264.7 after knockdown of α7nAChR and then activation of α7nAChR in LPS environment. Scale Bar: 100 µm. ^*^ *p* <0.05; ^**^ *p* <0.01; ^***^ *p* <0.001; ^****^ *p* <0.0001.

Next, we aimed to explore the mechanism of EA for LIP + SD. First we collected periodontal tissues from five groups to prepare single cell suspensions for flow cytometry (**Figure** **5**a). The results showed that the proportions of neutrophils, macrophages, and newly recruited macrophages increased in the disease group, while the proportions were decreased after EA; dendritic cells and monocytes showed no significant changes (Figure 5b; Figure S5a–d, Supporting Information). We then looked further at the proportions of α7nAChR^+^ cells in neutrophils, macrophages and newly recruited macrophages (Figure 5c) and found that only on macrophages did α7nAChR^+^ cells decline in the LIP + SD group relative to the LIP group, while they increased after EA (Figure 5d; Figure S5e,f, Supporting Information), suggesting that EA upregulates α7nAChR specifically in macrophages. Then, immunofluorescence staining of gingiva confirmed this trend (Figure 5e).

At last, we validated the role of macrophage α7nAChR in vitro. We supplemented exogenous ACh with LPS stimulation before administering GTS‐21 and MLA in culture, respectively. The results showed a significant decrease in the expression of pro‐inflammatory factors IL‐1β, IL‐6, and iNOS after administration of ACH and/or GTS‐21, whereas no significant changes were observed after administration of MLA, compared to LPS stimulation alone (Figure 5f). In addition, we also collected cell supernatants for ELISA and found decreases in the pro‐inflammatory factors IL‐1β, IL‐6, and TNF‐α after administration of ACh and/or GTS‐21, with no significant changes in IL‐10 (Figure S5g–j, Supporting Information). We also found an inhibition of M1 polarisation and promotion of M2 polarisation in macrophages after administration of ACh and/or GTS‐21, while the opposite trend was observed after administration of MLA (Figure 5g). Finally, we reduced α7nAChR on macrophages by α7nAChR knockdown on macrophages before administration of ACh and/or GTS‐21. We found that after administration of ACh or GTS‐21 alone caused a non‐significant decrease in the pro‐inflammatory factors IL‐1β, IL‐6, and iNOS, whereas their combination significantly reduced pro‐inflammatory factors, and there was an inhibition of M1‐polarization and a promotion of M2‐polarization in macrophages (Figure 5h,i). The above data suggest that EA inhibits the inflammatory response by activating α7nAChR on macrophages.

**Figure 6:**
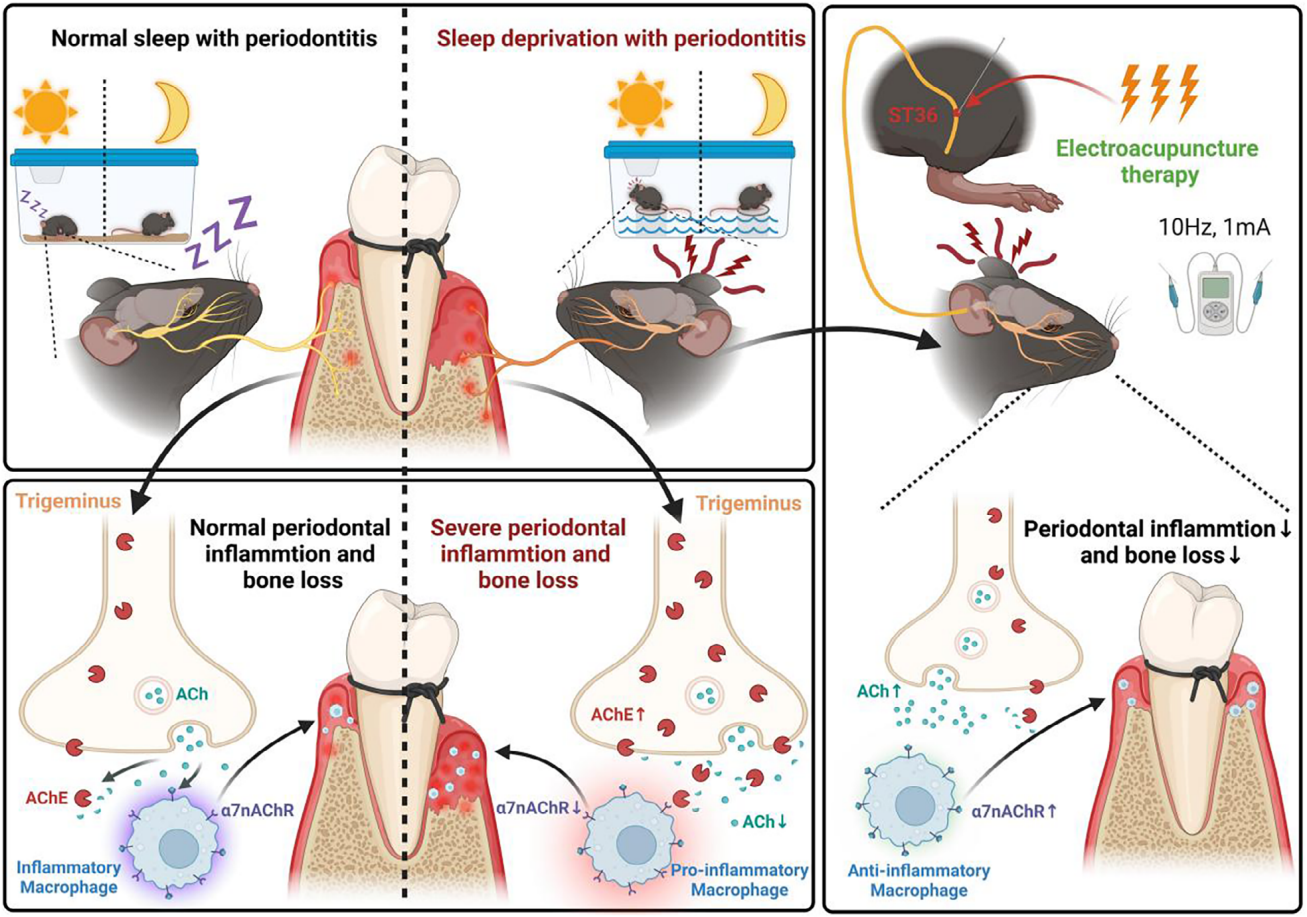
Schematic representation of SD aggravates LIP through trigeminal‐periodontal neuroimmune pathway mediated by the AChE‐ACh‐α7nAChR axis and EA ameliorates LIP + SD through the activation of ACh‐α7nAChR.

## 3 Discussion

This study investigated the impact of SD on LIP and the therapeutic potential of EA in SD + LIP mice, revealing that SD exacerbates alveolar bone resorption and inflammatory responses, while EA effectively reversed these pathological changes by reducing bone loss and suppressing pro‐inflammatory cytokines (**Figure** 6). Mechanistically, SD suppressed CAP function in periodontal tissues through triad dysregulation: 1) ACh depletion, 2) macrophage α7nAChR downregulation, and 3) trigeminal ganglion‐derived overexpression, whereas EA activated CAP to exert therapeutic effects—an outcome abolished by α7nAChR inhibitor MLA. Under inflammatory conditions, α7nAChR activation is modulated by the ACh/AChE balance.^[^ ^30^, ^31^ ^]^ Enhanced AChE activity reduced ACh levels, impairing α7nAChR binding and CAP activation.^[^ ^32^ ^]^ Crucially, AChE‐AAV injection into trigeminal ganglia attenuated bone resorption, confirming trigeminal‐originated AChE's role in SD‐driven pathology. Clinical validation in periodontitis patients with poor sleep (PSQI≥5) further demonstrated salivary/serum ACh reduction and AChE upregulation, supporting EA‐mediated α7nAChR activation as a novel neuroimmunomodulatory for comorbid sleep‐periodontitis conditions.

Emerging evidence underscores critical neuroimmune crosstalk in sleep disorder‐associated peripheral inflammation, with α7 nicotinic acetylcholine receptor (α7nAChR) downregulation emerging as a central pathogenic mechanism. Supporting our findings, Wei Wu et al. demonstrated that SD downregulates α7nAChR in microglia and astrocytes, suppresses the PI3K/AKT/GSK‐3β pathway, and exacerbates neuroinflammation and oxidative stress.^[^ ^12^ ^]^ Similarly, Jian‐jun Xue et al. showed that perioperative paradoxical SD reduces spinal α7nAChR in rats, aggravating postoperative hyperalgesia.^[^ ^33^ ^]^ These studies collectively establish α7nAChR deficiency as a hallmark of sleep‐related inflammatory amplification. Furthermore, sleep deprivation elevates AChE activity in brain regions, accelerating ACh metabolism.^[^ ^34^, ^35^, ^36^ ^]^ Building on this foundation, our study is the first to delineate the role of the CAP in sleep disorder‐aggravated periodontitis. Specifically, SD enhances trigeminal nerve‐derived AChE transmission to periodontal tissues, disrupting the AChE/ACh equilibrium, impairing α7nAChR activation, and ultimately suppressing CAP‐mediated anti‐inflammatory responses.

EA demonstrates robust therapeutic efficacy across diverse pathologies through α7nAChR‐mediated CAP modulation. Yang et al. reported that EA inhibited postoperative ileus inflammation through α7nAChR‐mediated activation of the JAK2/STAT3 signaling pathway.^[^ ^21^ ^]^ Similarly, Liu et al. revealed that EA mitigates brain injury after asphyxial cardiac arrest through α7nAChR‐dependent suppression of microglial NLRP3 inflammasome activation.^[^ ^37^ ^]^ Our prior study established EA's efficacy in alleviating periodontitis through immune homeostasis and modulating periodontal microbiota composition.^[^ ^20^ ^]^ Extending these findings, the current study confirms that EA exerts anti‐inflammatory effects via α7nAChR activation on periodontal macrophages — effects abolished by the α7nAChR antagonist MLA. These collective data suggest EA restores periodontal immune homeostasis through systemic modulation of cholinergic signaling, potentially involving neuroimmune crosstalk.

The oral‐brain axis represents a bidirectional neuroimmune network where CNS dysregulation exacerbates oral pathologies through cholinergic imbalance.^[^ ^38^, ^39^ ^]^ For instance, P.S. Thrane et al. demonstrated that HPA axis hyperresponsiveness in rats increases susceptibility to periodontitis.^[^ ^40^, ^41^, ^42^, ^43^ ^]^ Similarly, T. Myhrer et al. reported that hippocampal lesion‐induced dysregulation of neuroendocrine‐immune networks amplifies periodontal tissue destruction.^[^ ^44^ ^]^ Our study further reveals that SD promotes periodontitis progression via trigeminal ganglion‐derived AChE transmission, which suppresses the periodontal CAP. Crucially, EA at ST36 restores ACh homeostasis and reactivates CAP via α7nAChR upregulation, establishing a translational paradigm for comorbid neurological‐oral disorders. These findings not only elucidate neuroimmune crosstalk within the oral‐brain axis but also propose novel therapeutic strategies for comorbid neurological and oral inflammatory disorders.

Our study advances the understanding of sleep disorders in periodontal pathogenesis through three novel discoveries: While prior research established epidemiological links between sleep disorders and periodontitis,^[^ ^45^, ^46^, ^47^, ^48^ ^]^ we elucidated the mechanistic axis via in vivo/in vitro models demonstrating that SD activates trigeminal ganglion‐derived AChE transmission, disrupting the ACh/α7nAChR balance and suppressing CAP function. Second, this study first demonstrated the efficacy of EA therapy based on neuroimmune regulation in treating periodontitis with sleep disorders, offering a safe, minimally invasive therapeutic option for clinically relevant patients. Finally, this study elucidates the critical role of the “oral‐brain axis” in the occurrence, development, and treatment of periodontitis associated with systemic diseases, as well as the pivotal role of the cholinergic pathway (AChE, ACh, α7nAChR) in this pathology.

However, there are some limitations to this study. First, in the animal model, we used a combined approach of modified multiple‐platform sleep deprivation protocol and the silk ligation experimental periodontitis method to establish the disease model, which has limitations, including greater stress on mice, water contamination, and the inability to fully simulate the health problems that may be caused by sleep deprivation in humans, etc. Second, the differential effects of GTS‐21 application in the in vivo and in vitro experiments may be due to the fact that low baseline α7nAChR expression in the periodontium of SD + LIP mice may limit agonist efficacy appreciably. Thirdly, this study focused on the neuroimmune mechanisms underlying SD‐mediated regulation of LIP, without addressing other potential contributors such as the HPA axis, oxidative stress, oral microbiota, and sympathetic nervous system, which may synergistically participate in SD‐driven modulation of periodontitis. Fourthly, the specific dosage, frequency, and long‐term effects of EA treatment require further investigation to optimize its therapeutic potential in this context. Finally, due to the lack of direct genetic manipulation, real‐time monitoring of ACh dynamics, and unresolved upstream regulatory mechanisms, the role of the AChE‐ACh‐α7nAChR axis in periodontitis remains incompletely elucidated.

## 4 Conclusion

In summary, this study reports the role of the AChE‐ACh‐α7nAChR axis in the modulation of inflammatory responses and neuroimmune interactions in periodontal disease in a sleep deprivation model and EA targeting this pathway. Our data show that SD inhibits the cholinergic anti‐inflammatory pathway and thus periodontitis by increasing trigeminal ganglion‐derived AChE in periodontal tissues, decreasing ACh binding to α7nAChR, while EA activates the cholinergic anti‐inflammatory pathway and partially reverses this effect. The results of this study elucidate the mechanism by which sleep deprivation affects peripheral nerve inflammation, which is conducive to the targeted treatment of sleep deprivation‐induced periodontitis.

## 5 Experimental Section

### NHANES Data Analysis

The population cohort analyzed in this study was sourced from the “Examination Data” and “Questionnaire Data” of the NHANES database (https://wwwn.cdc.gov/nchs/nhanes) spanning from 2009 to 2014. The “Examination Data” section encompasses “Oral Health ‐ Periodontal” data for the population, while the “Questionnaire Data” module covers “Sleep Disorders” data. The required data were read and integrated using the “foreign” package in R. Clinical attachment loss (CAL), probing depth (PD), and the presence of sleep disorders were statistically analyzed for the cohort. Based on whether individuals had sleep disorders, they were divided into healthy controls and a sleep disorder group, and the CAL and PD between the two groups were compared.

### Clinical Human Samples Collection

The collection of clinical samples in this study was approved by the Ethics Committee of the College of Stomatology, Chongqing Medical University [NO:2022 (LSNo.099)]. Fifteen volunteers were categorized into three groups based on periodontal examination indices and the Pittsburgh Sleep Quality Index (PSQI) questionnaire results: the CTR group (healthy control group, 5 cases without periodontitis [bleeding on probing (BOP) positive and probing depth (PD) > 5 mm] and PSQI < 5), the PD group (periodontitis group without sleep disturbance, 5 cases with periodontitis and PSQI < 5), and the PS group (periodontitis with sleep disturbance group, 5 cases with periodontitis and PSQI ≥ 5). Standard gingival punch biopsies were collected from the volunteers under local anesthesia; gingival tissue samples from healthy individuals were obtained during crown lengthening surgery, while gingival tissue samples from periodontitis patients were collected prior to the extraction of hopeless teeth. Additionally, saliva and blood samples were collected from all volunteers.

### Animals and Experimental Procedures

The protocols were reviewed and approved by the Ethics Committee of the College of Stomatology, Chongqing Medical University [NO:2023 (LSNo.065)]. Specific pathogen‐free (SPF) C57BL/6 mice were housed in the Animal Facility at the College of Stomatology, Chongqing Medical University. All the mice had free access to food and water. Eight‐week‐old mice were randomised into five groups: CTR (control); LIP (ligature‐induced periodontitis); LIP + SD (LIP with sleep deprivation); LIP + EA (LIP treated with electroacupuncture stimulation); LIP + SD + EA (LIP + SD treated with EA).

The mice underwent LIP modeling after 7 days of adaptive feeding. Periodontitis was induced by ligature placement. Mice were anesthetized with Delivector Avertin (Dowobio, China). Then, 5‐0 silk ligatures (Ethicon, USA) were placed around the cervical region of the bilateral maxillary second molars.

For sleep deprivation, mice were placed in a modified multi‐platform water tank at 8:00 AM each day for 2 h of training for three consecutive days after LIP. Starting on day 12, mice were subjected to SD for 7 consecutive days. Each water tank contained 12 platforms and housed 6 mice. Each mouse in the water tank could move from one platform to another by jumping. The water bath was filled to a depth of 4 cm below the platform. When mice entered the rapid eye movement (REM) sleep stage — a paradoxical phase characterized by muscle relaxation that could cause them to fall into the water — they would awaken and attempt to climb onto the platform. The mice then woke up and tried to climb onto the platform to avoid drowning. Throughout the experiment, the water was changed every morning at 8:00 AM to keep the tank clean.

Starting from day 12, mice received EA (10 Hz, 1 mA, square wave) by acupoint nerve stimulator (Suzhou Huize Medical Technology Co., Ltd Model:ZR‐TY‐301, China) at 10:00 AM zeitgeber time, 20 min time^−1^, every other day for 7 days. EA was performed as described in previous studies.^[^ ^20^ ^]^ Briefly, the mice were immobilized and exposed to the hind limb area with a custom‐made binding device without violent struggle. EA was delivered using 0.3‐mm diameter sterilized disposable stainless‐steel acupuncture needles, bilaterally inserted as deep as 3 mm at acupoint Zusanli (ST36). The ST36 acupoint was in the tibialis anterior muscle and 3 mm below the knee joint. At the same time, control mice received sham EA, by inserting needles into 3‐mm depth at the midpoint of the tail, a position that did not correspond to any traditional acupoint and was given the same electrical stimulation. After EA treatment, the mice were immediately placed back in the modified multi‐platform water tank. The weight changes of the mice were recorded throughout the operation.

All mice were sacrificed under anesthesia using Delivector Avertin at the end of the experiment (10:00 AM on the 19th day), and plasma, periodontal tissues, and trigeminal ganglia were collected at the same time point. Serum neurotransmitters (dopamine, epinephrine, norepinephrine, acetylcholine) were detected by ELISA. The test samples were serum that was immediately isolated and frozen after sacrifice.

### Animal Behavior Experiments

The open field test (OFT) and the elevated plus maze (EPM) experiments were used as ancillary experiments to detect successful sleep deprivation. The open field consisted of a square enclosure with a white wooden floor. The elevated plus maze consisted of two opposing closed arms with identically sized 50‐cm high walls, two open arms, and a central platform at the intersection of the open and closed arms. The plus maze was elevated 50 cm above the ground. At the start of the test, the mouse was placed in the centre of the open field or maze and allowed 5 min of free exploration. During these 5 min of exploration, the video tracking software recorded the relevant parameters.

### ELISA

Corticosterone, dopamine (DA), epinephrine (EPI), noradrenaline (NE), acetylcholine (ACh), acetylcholinesterase (AChE), interleukin 1β (IL‐1β), interleukin 6 (IL‐6), the tumor necrosis factor α (TNF‐α) and interleukin 10 (IL‐10) levels in serum, periodontal tissue suspension and cell supernatant were measured by enzyme‐linked immunosorbent assay (ELISA; Yingxin, China). These samples were stored at −80 °C until assays were performed. Neurotransmitters and cytokines were analyzed by ELISA according to the manufacturers’ protocol.

### Quantification of Maxillary Alveolar Bone Resorption

The maxillaries were scanned using a high‐resolution CT system (Skyscan 1172; Skyscan, Aartselaar, Belgium) and reconstructed by applying 3D Slicer software. The linear distance from the cementoenamel junction (CEJ) to the alveolar bone crest (ABC) of the second molar was measured from the mesial and distal sides. The CEJ‐ABC values were recorded for each group (n = 6–8 per group for all micro‐CT analyses).

### Histological Assays

For tartrate‐resistant acid phosphatase (TRAP) staining, the maxillaries were decalcified in 0.5 m EDTA, embedded in paraffin, and sectioned into 5‐µm thickness. TRAP staining was performed using a commercial kit (APExBIO, USA).

For tissue immunofluorescence (IF) staining, the sections were then incubated with hyaluronidase (2 mg mL^−1^; Sigma, Germany) for 1 h at 37 °C for antigen retrieval. After blocking with 1% BSA (Beyotime, China), the sections were incubated overnight at 4 °C with F4/80, CD3, CD19, PGP9.5, CD31, and CD90 primary antibodies (Proteintech, USA), respectively. Afterward, the sections were incubated with corresponding secondary antibodies for 1 h and then counterstained with DAPI (Beyotime). Images were captured with a high‐resolution digital microscope (Nikon, Japan).

### Quantitative Real‐Time PCR

The periodontal tissue samples were collected and frozen for grinding, and RNA was extracted using an RNAeas Animal RNA Isolation Kit with Spin Column (Beyotime). qPCR was performed on the CFX96 Touch Real‐Time PCR Detection System (Bio‐Rad, USA) using SYBR Green reagent (Roche, Switzerland). Primer sequences used in this study can be found in Table S1 (Supporting Information).

### Western Blot (WB)

Standard Western blot techniques were used on the periodontal tissues. The membranes were blocked with 5% skimmed milk and incubated with primary antibodies (α7nAChR, 1:500; GAPDH, 1:1000; Santa Cruz, USA). Anti‐rat IgG (1:1000) and anti‐rabbit IgG (1:1000) were used for secondary antibodies (Thermo Fisher Scientific, USA).

### Pharmacological Intervention

α7nAchR agonist GTS‐21 and antagonists Methyllycaconitine citrate (MLA) was purchased from MCE (UK). The dose was based on early studies. Mice received an i.p. injection of 4 mg kg^−1^ GTS‐21, 6 mg kg^−1^ MLA or PBS daily for 7 days with or without EA.

### Flow Cytometry

Single‐cell suspensions from periodontal tissue and blood were prepared according to a previously published method.^[^ ^28^, ^29^ ^]^ Blood samples were collected from each group via cardiac puncture into EDTA‐lined tubes at the end of treatment. Red blood cells were lysed using Red Blood Cell Lysis Buffer (Abcam, UK), and then the white cells were pelleted by centrifugation at 400× g for 5 min at 4 °C. Gingival tissues were excised from the buccal and palatal sides of the three molars, digested with type II and IV collagenase and DNase, and mashed through cell strainers. Flow cytometry was performed using a BD FACSCanto II flow cytometer (BD Biosciences, USA). Single‐cell suspensions were stained with the antibodies listed in Table S2 (Supporting Information) and blocked with mouse anti‐CD16/CD32 (BD Biosciences). It was distinguished by the expression of CD11c, CD11b, CD45, CD64, CD86, F4/80, MHCII, Ly6G, Ly6C, and α7nAChR for detecting myeloid cells. For α7nAChR, an indirect method was employed for single‐cell staining. The results were analyzed using FlowJo and Cytoexpert software.

### Clodronate‐induced macrophage depletion

For all the experiments involving clodronate liposomal administration in this study, 5 mg kg^−1^ of clodronate liposomes (LIPOSOMA, Netherlands) were intraperitoneally injected into mice. Likewise, 5 mg kg^−1^ of control liposomes were similarly injected. Starting on day 6, clodronate or control liposomes were administered on days 8, 10, 12, 14, 16, and 18 as per the study design.

### Cell Culture

The macrophage cell line RAW264.7 (Procell, China) and neuronal cell line HT22 (Procell) were used in our study. All cell lines were cultured at 37 °C in a 5% CO_2_ incubator with a culture medium composed of high‐glucose DMEM (Gibco, USA) and 10% fetal bovine serum (Gibco), and the culture medium was renewed every 2  days.

For the co‐culture experiments, AChE in HT22 was overexpressed using the plasmid (Tsingke, China), and then transwell co‐cultured and direct contact co‐cultured with RAW264.7 according to certain ratios, respectively, and then used for flow cytometry assay after 48 h.

For RAW264.7 separate incubation experiments, cells were stimulated with lipopolysaccharide (LPS, 500 ng mL^−1^; Sigma) to establish an inflammatory environment. Subsequently, further incubation was performed for 24–48 h in different experiments using the indicated concentrations of AChE recombinant protein, exogenous ACh (Acetylcholine chloride, MCE), GTS‐21, and MLA. Then, the cells were washed once in PBS, fixed in the fixative for 15 min, washed three times, added to the immunostaining blocking solution, and blocked at 37°C for 1 h. Thereafter, the CD86 and CD206 primary antibody (Proteintech) was incubated and washed three times, and the secondary antibody was incubated, washed, incubated with DAPI stain, washed, and sealed with blocker. At last, the slices were photographed by a laser confocal microscope (Leica TCS SP8, Vizsla, Germany).

### Microinjection into the Trigeminal Ganglion

To selectively ablate AChE expression in trigeminal ganglion, adeno‐associated virus (AAV;Tsingke) encoding inhibitory designer receptor exclusively activated by AChE (mCherry) or control green fluorescent protein (eGFP) was directly injected into trigeminal ganglion through the infraorbital foramen. The trigeminal ganglion was collected to observed the expression of AChE‐AAV/GFP‐AAV autofluorescence by electron microscopy.

### Statistical Analysis

All data were statistically analysed by Prism 9 (Graphpad, USA) and presented as mean ± SEM. The data were analyzed using Student's *t* tests and 1‐way or 2‐way ANOVA followed by post hoc tests. Differences of *p* <0.05 were considered significant in all statistical analyses. Statistically significant differences are shown with asterisks as follows: ^*^ *p* <0.05, *^*^ *p* <0.01, **^*^ *p* <0.001, ***^*^ *p* <0.0001, and ns for no significance.

## Conflict of Interest

The authors declare no conflict of interest.

## Supporting information

Supporting Information

## Data Availability

The data that support the findings of this study are available from the corresponding author upon reasonable request.

## References

[1] N. Meyer , A. G. Harvey , S. W. Lockley , D. J. Dijk , Lancet 2022, 400, 1061. 36115370 10.1016/S0140-6736(22)00877-7

[2] L. Korostovtseva , M. Bochkarev , Y. Sviryaev , Sleep Med. Clin. 2021, 16, 485. 34325825 10.1016/j.jsmc.2021.05.001

[3] M. Bishir , A. Bhat , M. M. Essa , O. Ekpo , A. O. Ihunwo , V. P. Veeraraghavan , S. K. Mohan , A. M. Mahalakshmi , B. Ray , S. Tuladhar , S. Chang , S. B. Chidambaram , M. K. Sakharkar , G. J. Guillemin , M. W. Qoronfleh , D. M. Ojcius , BioMed. Res. Int. 2020, 2020, 5764017. 33381558 10.1155/2020/5764017 PMC7755475

[4] H. Jin , Y. Zhang , R. Ren , Y. Shi , X. Feng , M. V. Vitiello , X. Tang , Sleep Breath. 2024, 29, 16. 39601918 10.1007/s11325-024-03202-1

[5] V. Grover , R. Malhotra , H. Kaur , J. Indian Soc. Periodontol 2015, 19, 304. 26229272 10.4103/0972-124X.154173 PMC4520116

[6] J. S. Park , Y. Jeong , J. Jung , J. J. Ryu , H. K. Lim , S. K. Jung , I. S. Song , J. Clin. Periodontol. 2021, 48, 1066. 34101218 10.1111/jcpe.13508

[7] M. R. Opp , L. Imeri , Acta Neurobiol Exp (Wars) 1999, 59, 45. 10230076 10.55782/ane-1999-1295

[8] J. A. Dani , Biol. Psychiatry 2001, 49, 166. 11230867 10.1016/s0006-3223(00)01011-8

[9] B. Bonaz , V. Sinniger , S. Pellissier , J. Physiol. 2016, 594, 5781. 27059884 10.1113/JP271539 PMC5063949

[10] L. Rueda Ruzafa , J. L. Cedillo , A. J. Hone , Int. J. Environ. Res. Public Health 2021, 18, 1189. 33572734 10.3390/ijerph18031189 PMC7908252

[11] N. Zoheir , D. F. Lappin , C. J. Nile , Inflamm. Res. 2012, 61, 915. 22777144 10.1007/s00011-012-0513-z PMC3786254

[12] R. Xue , Y. Wan , X. Sun , X. Zhang , W. Gao , W. Wu , Front Immunol. 2019, 10, 2546. 31736967 10.3389/fimmu.2019.02546 PMC6828928

[13] M. R. Irwin , Nat. Rev. Immunol. 2019, 19, 702. 31289370 10.1038/s41577-019-0190-z

[14] G. Hajishengallis , Nat. Rev. Immunol. 2015, 15, 30. 25534621 10.1038/nri3785 PMC4276050

[15] W. Pan , Q. Wang , Q. Chen , Int. J. Oral. Sci. 2019, 11, 30. 31685798 10.1038/s41368-019-0064-z PMC6828663

[16] L. Ulloa , Nature 2021, 598, 573. 34646023 10.1038/d41586-021-02714-0 PMC9628694

[17] X. Yin , W. Li , T. Liang , B. Lu , H. Yue , S. Li , V. W. Zhong , W. Zhang , X. Li , S. Zhou , Y. Mi , H. Wu , S. Xu , JAMA Netw. Open 2022, 5, 2220563. 10.1001/jamanetworkopen.2022.20563 PMC9264041 35797047

[18] Z. Peng , M. Ying , L. Meijun , Y. U. Di , L. Huiying , W. Fuchun , X. U. Xiaohong , J. Tradit. Chin. Med. 2024, 44, 974. 39380228 10.19852/j.cnki.jtcm.20240806.002 PMC11462537

[19] M. R. Lisboa , D. V. Gondim , E. Ervolino , M. L. Vale , N. P. Frota , N. L. Nunes , V. C. Mariguela , T. M. Jr , M. R. Messora , F. A. Furlaneto , J. Periodontol. 2015, 86, 801. 25741581 10.1902/jop.2015.140630

[20] K. Liu , L. Yang , X. Wang , Q. Huang , K. Tuerhong , M. Yang , R. Zhang , Y. Li , S. Yang , J. Clin. Periodontol. 2023, 50, 368. 36356944 10.1111/jcpe.13748

[21] N. N. Yang , J. W. Yang , Y. Ye , J. Huang , L. Wang , Y. Wang , X. T. Su , Y. Lin , F. T. Yu , S. M. Ma , L. Y. Qi , L. L. Lin , L. Q. Wang , G. X. Shi , H. P. Li , C. Z. Liu , Theranostics 2021, 11, 4078. 33754049 10.7150/thno.52574 PMC7977469

[22] N. Ding , Q. Wei , W. Deng , X. Sun , J. Zhang , W. Gao , Oxid. Med. Cell Longev. 2021, 2021, 6673610. 33897942 10.1155/2021/6673610 PMC8052151

[23] J. Deng , C. Lu , Q. Zhao , K. Chen , S. Ma , Z. Li , J. Periodontal Res. 2022, 57, 246. 34878170 10.1111/jre.12958

[24] R. Torres‐Rosas , G. Yehia , G. Peña , P. Mishra , R. del , M. Thompson‐Bonilla , M. A. Moreno‐Eutimio , L. A. Arriaga‐Pizano , A. Isibasi , L. Ulloa , Nat. Med. 2014, 20, 291. 24562381 10.1038/nm.3479 PMC3949155

[25] G. Xu , Z. Duanmu , Q. Yin , Zhen Ci Yan Jiu 1993, 18, 1. 8082273

[26] E. Nizri , I. Wirguin , T. Brenner , Drug News Perspect. 2007, 20, 421. 17992264 10.1358/dnp.2007.20.7.1149629

[27] X. Li , M. Kiprowska , T. Kansara , P. Kansara , P. Li , J. Dent. Res. 2022, 101, 1441. 35708472 10.1177/00220345221102084 PMC9608094

[28] K. L. Cole , G. J. Seymour , R. N. Powell , J. Periodontol. 1987, 58, 569. 2957490 10.1902/jop.1987.58.8.569

[29] A. R. Kim , E. J. Bak , Y. J. Yoo , J. Periodontal Res. 2022, 57, 332. 34927238 10.1111/jre.12963

[30] K. T. Dineley , A. A. Pandya , J. L. Yakel , Trends Pharmacol. Sci. 2015, 36, 96. 25639674 10.1016/j.tips.2014.12.002 PMC4324614

[31] Y. Takada‐Takatori , T. Kume , Y. Izumi , Y. Ohgi , T. Niidome , T. Fujii , H. Sugimoto , A. Akaike , Biol. Pharm. Bull. 2009, 32, 318. 19252271 10.1248/bpb.32.318

[32] R. Benfante , S. Di Lascio , S. Cardani , D. Fornasari , Aging Clin. Exp. Res. 2021, 33, 823. 31583530 10.1007/s40520-019-01359-4

[33] Y. Y. Cui , Z. Q. Xu , X. Y. Qin , H. J. Hou , J. Zhang , J. J. Xue , IBRO Neurosci. Rep. 2024, 17, 389. 39559484 10.1016/j.ibneur.2024.10.002 PMC11570470

[34] R. M. Lataro , C. A. Silva , C. Tefé‐Silva , C. M. Prado , H. C. Salgado , Am. J. Hypertens. 2015, 28, 1201. 25758777 10.1093/ajh/hpv017

[35] G. Budryn , I. Majak , J. Grzelczyk , D. Szwajgier , A. Rodríguez‐Martínez , H. Pérez‐Sánchez , Nutrients. 2022, 14, 2476. 35745206 10.3390/nu14122476 PMC9227119

[36] A. Bhide , A. Sen , Phys. Chem. Chem. Phys. 2023, 25, 21149. 37551603 10.1039/d3cp01480k

[37] Y. Liu , L. Zhang , R. Han , W. Bai , J. Li , C. Gao , J. Neuroimmunol. 2022, 367, 577873. 35487121 10.1016/j.jneuroim.2022.577873

[38] Y. Wu , Y. Lan , J. Mao , J. Shen , T. Kang , Z. Xie , Int. J. Oral Sci. 2023, 15, 34. 37580325 10.1038/s41368-023-00241-4 PMC10425412

[39] S. Malan‐Müller , R. Vidal , E. O'Shea , E. Montero , E. Figuero , I. Zorrilla , J. de Diego‐Adeliño , M. Cano , M. P. García‐Portilla , A. González‐Pinto , J. C. Leza , Transl. Psychiatry 2024, 14, 419. 39368974 10.1038/s41398-024-03122-4 PMC11455920

[40] C. D'Mello , M. G. Swain , Curr. Top. Behav. Neurosci. 2017, 31, 73. 27677781 10.1007/7854_2016_37

[41] A. Kantarci , C. M. Tognoni , W. Yaghmoor , A. Marghalani , D. Stephens , J. Y. Ahn , I. Carreras , A. Dedeoglu , Sci. Rep. 2020, 10, 18561. 33122702 10.1038/s41598-020-75517-4 PMC7596239

[42] B. S. Main , M. R. Minter , Front. Neurosci. 2017, 11, 151. 28386215 10.3389/fnins.2017.00151 PMC5362619

[43] A. Mullard , Nature 2021, 595, 162. 34193994 10.1038/d41586-021-01763-9

[44] T. Breivik , P. S. Thrane , P. Gjermo , A. Cools , T. Myhrer , J. Periodontal Res. 2002, 37, 360. 12366859 10.1034/j.1600-0765.2002.00366.x

[45] M. C. Carra , P. Balagny , P. Bouchard , Periodontol 2000 2024, 96, 42. 39233377 10.1111/prd.12611 PMC11579834

[46] M. Iwasaki , M. Usui , W. Ariyoshi , K. Nakashima , Y. Nagai‐Yoshioka , M. Inoue , K. Kobayashi , T. Nishihara , J. Clin. Periodontol. 2022, 49, 59. 34605052 10.1111/jcpe.13561

[47] A. A. Alhassani , A. l.‐Z. MS , PLoS One 2020, 15, 0234487. 10.1371/journal.pone.0234487 PMC7297327 32544165

[48] M. Romandini , G. Gioco , G. Perfetti , G. Deli , E. Staderini , A. Laforì , J. Clin. Periodontol. 2017, 44, 490. 28211083 10.1111/jcpe.12713

