# Supplementary material for: Sleep Deprivation Aggravates Periodontitis Through Trigeminal‐Periodontal Neuroimmune Pathway Mediated by the AChE‐ACh‐α7nAChR Axis
Source: Adv Sci (Weinh). 2025 Aug 14;12(43):e00945. doi: 10.1002/advs.202500945 (PMC12631922; doi:10.1002/advs.202500945)
Supplement: Supplementary file 1 — Supporting Information [file ADVS-12-e00945-s001.docx]

Sleep deprivation aggravates periodontitis through trigeminal-periodontal neuroimmune pathway mediated by the AChE-ACh-α7nAChR axis

SUPPORT INFOMATION


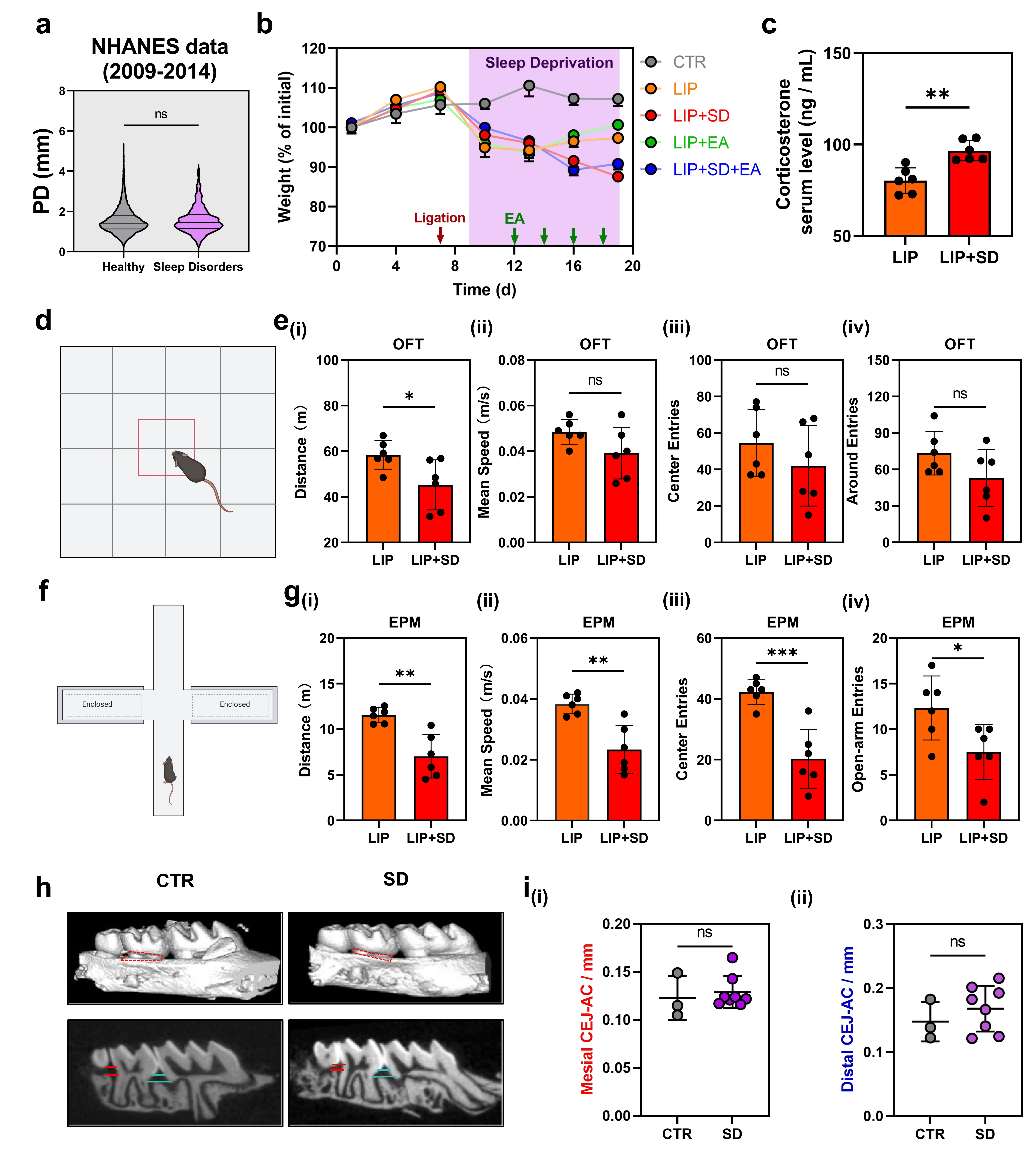


**Supplementary Figure 1** (a) Bar chart of probing depth (PD) in the healthy control group versus the sleep disorder group from the NHANES database between 2009 and 2014. (b) The body weight change chart of the whole experiment process. (c) Serum levels of corticosterone. (d) The diagram of the open field test. (e) Distance traveled (i), mean speed (ii), center entries (iii), and around entries (iv) in the open-field test. (f) The diagram of the elevated plus maze. (g) Distance traveled (i), mean speed (ii), center entries (iii), and around entries (iv) in the elevated plus maze. (h) 3D reconstruction of micro-CT scanned images of the alveolar bone. Scale bar = 1 mm. (i) Comparison of the mesial (i) and distal (ii) CEJ–ABC distances of alveolar bone loss. **P* < 0.05; ***P* < 0.01.


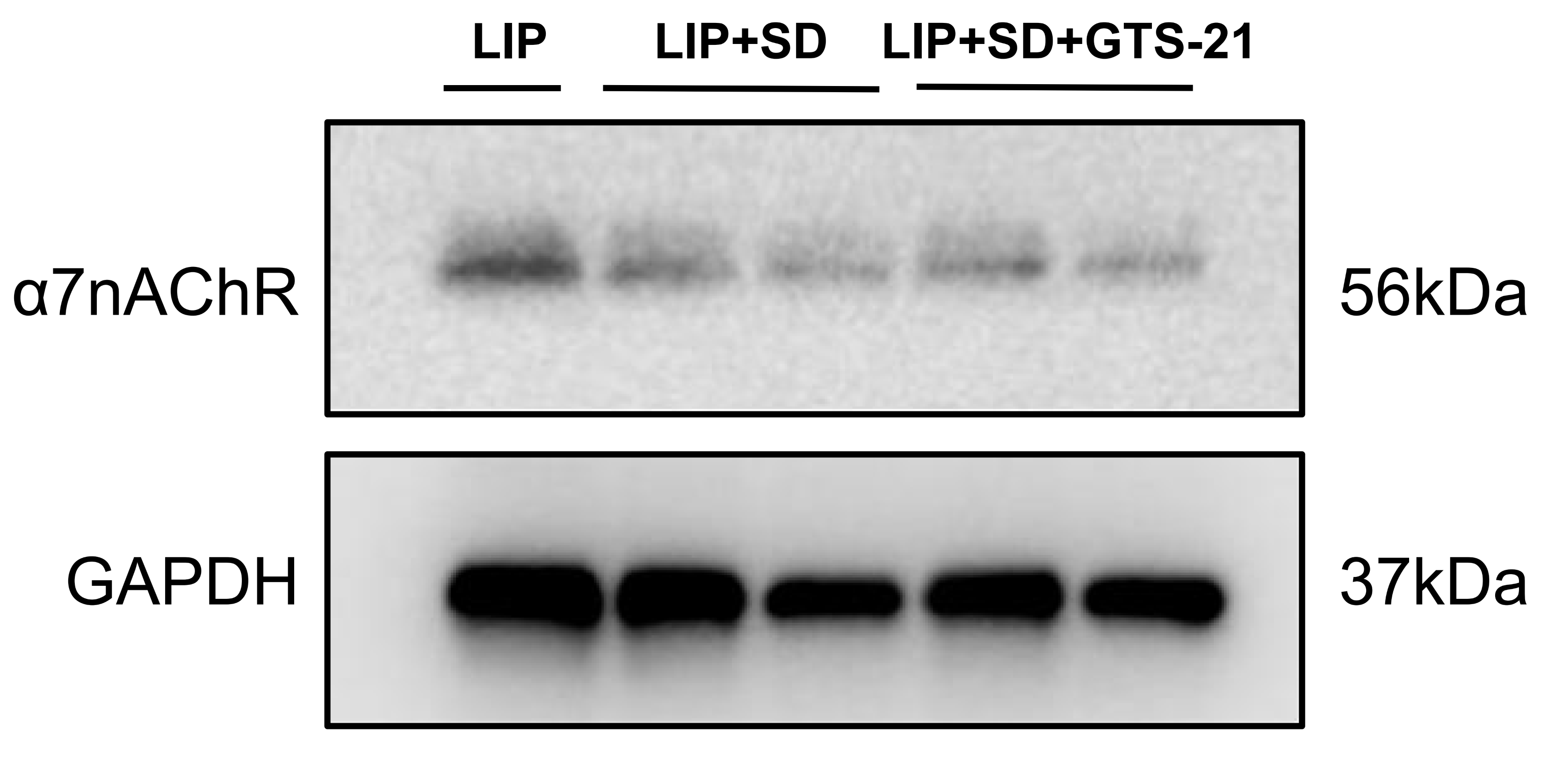


**Figure S2** Western blots of α7nAChR in periodontal tissue.


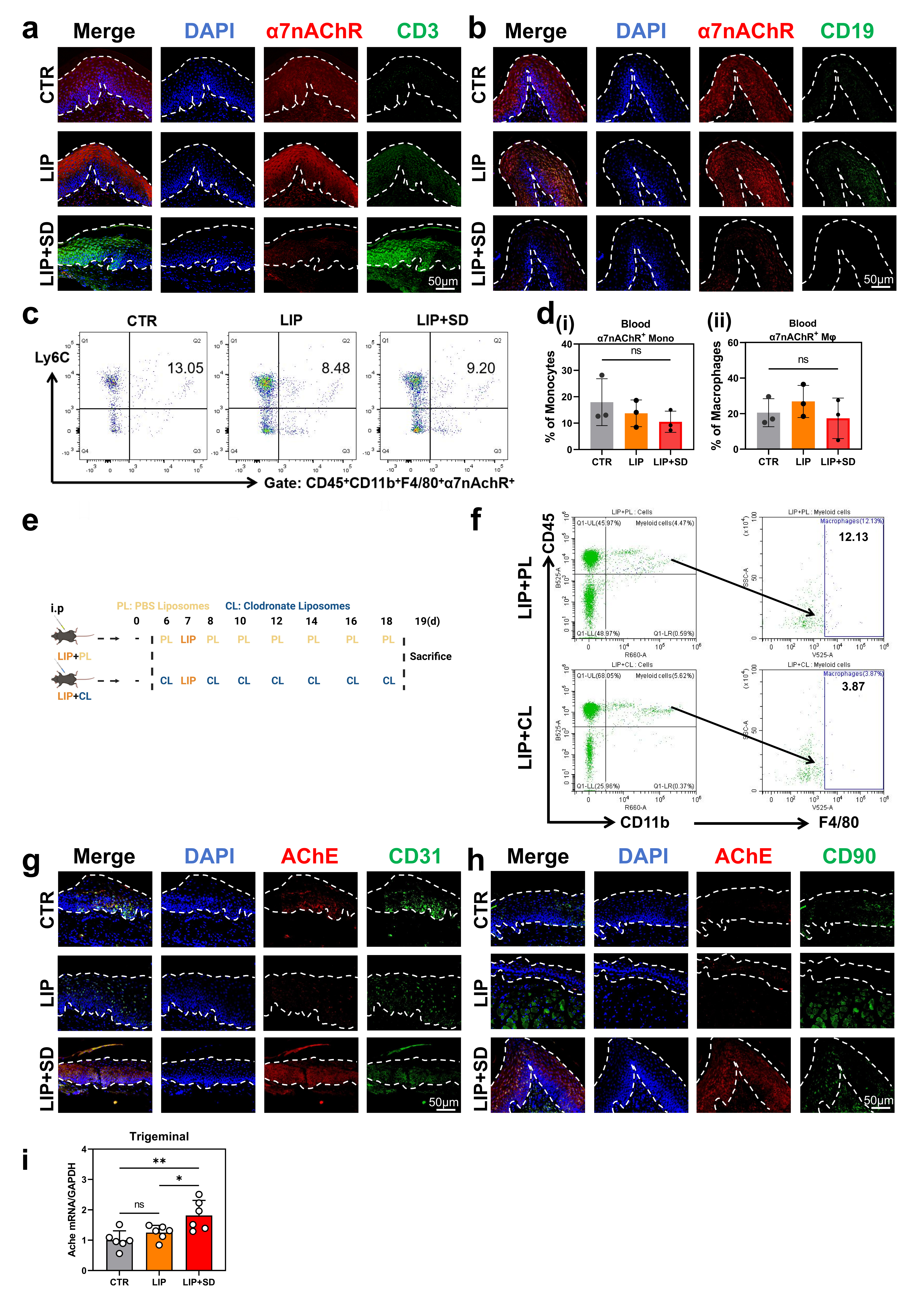


**Supplementary Figure 3** (a) Co-expression of α7nAChR and T cell marker CD3 in periodontal tissue. (b) Co-expression of α7nAChR and B cell marker CD3 in periodontal tissue (c) Flow cytometric gate of α7nAChR^+^ macrophages and monocytes in blood sample. (d) The frequencies of α7nAChR^+^ monocytes (i) and α7nAChR^+^ macrophages (ii) in blood sample. (e) Flow chart of chlorophosphate depletion macrophage experiment. (f) Flow cytometric gating strategy of macrophages in the chlorophosphate depletion macrophage experiment. (g) Co-expression of AChE and vascular endothelial marker CD31 in periodontal tissue. (h) Co-expression of AChE and cell adhesion marker CD90 in periodontal tissue. (i) Trigeminal ganglion mRNA expression of AChE. **P* < 0.05.


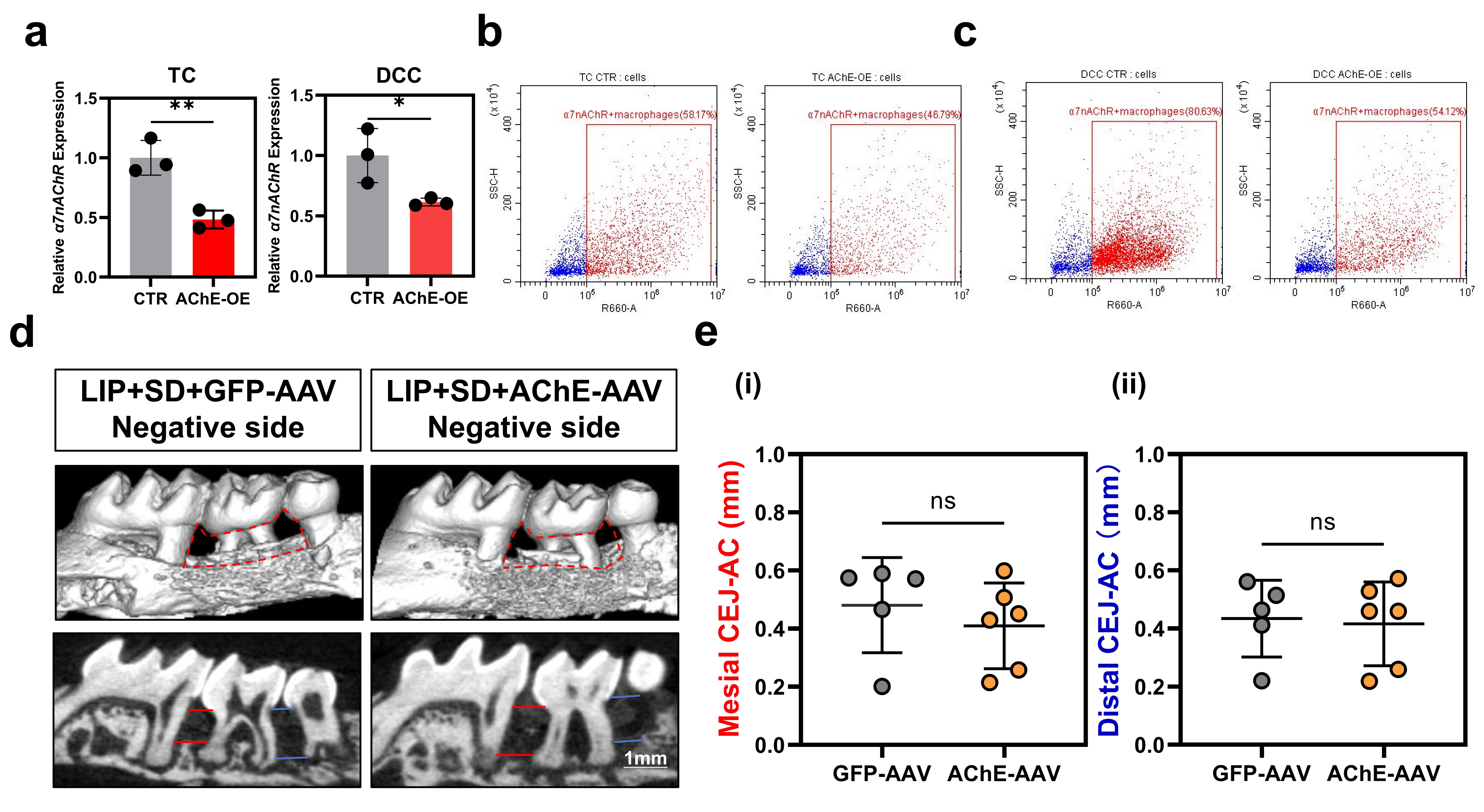


**Figure S4** Flow cytometric gate of α7nAChR^+^ macrophages in transwell co-culture (a) and direct contact co-culture (b). (c) 3D reconstruction of micro-CT scanned images of the alveolar bone. Scale bar = 1 mm. (d) Comparison of the mesial (i) and distal (ii) CEJ–ABC distances of alveolar bone loss. **P* < 0.05.


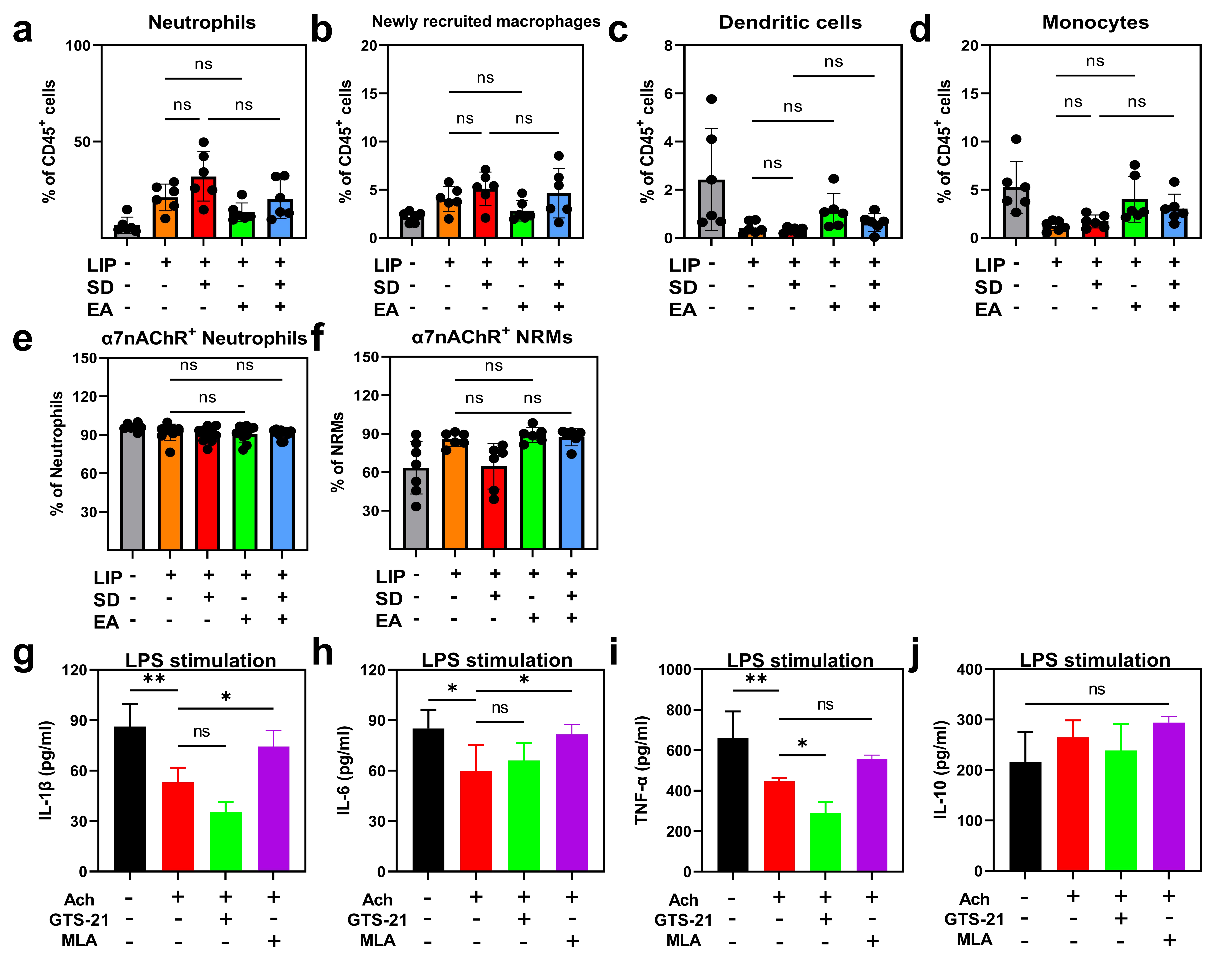


**Figure S5** The frequencies of neutrophils (a), newly recruited macrophages (b), dendritic cells (c), and monocytes (d). The frequencies of α7nAChR^+^ neutrophils (e) and α7nAChR^+^ newly recruited macrophages (f) in periodontal tissues. Cell supernatant levels of IL-1β (g), IL-6 (h), TNF-α (i), and IL-10 (j). ns not significant; **P* < 0.05; ***P* < 0.01.

Table S1 Primers for RT-PCR analysis

| **Gene** | **Direction** | **Primer** **(5’-3’)** |
| --- | --- | --- |
| GAPDH | F (forward) | GACATCAAGAAGGTGGTGAAGC |
|  | R (reverse) | GAAGGTGGAAGAGYGGGAGTT |
| TRAP | F | CTTGTGGACGAAAATATGTGGCT |
|  | R | GACTTTCCTGTCGAATGCACT |
| IL-1β | F | CACCTCTCAAGCAGAGCACAG |
|  | R | GGGTTCCATGGTGAAGTCAAC |
| IL-6 | F | GTTCTCTGGGAAATCGTGGA |
|  | R | TGTACTCCAGGTAGCTATGG |
| IL-17a | F | GAGAAGATGCTGGTGGGT |
|  | R | TTTGCTGAGAAACGTGGG |
| TNF-α | F | CCAAATGGCCTCCCTCTCAT |
|  | R | TGGTGGTTTGCTACGACGTG |
| IL-10 | F | GACCAGCTGGACAACATACTGCTAA |
|  | R | GATAAGGCTTGGCAACCCAAGTAA |
| iNOS | F | CCAACCTGCAGGTCTTCGATG |
|  | R | GTCGATGCACAACTGGGTGAAC |
| CHRM2 | F | CCCCAATACAGTGTGGACAA |
|  | R | GCAGGGTTGATGGTGCTATT |
| CHRNA7 | F | CCCTGGCTTTGCTGGTATT |
|  | R | GCATGAAGACAGTCAGAGAGAAAGTAA |
| CHRNB1 | F | TGAAGAAATGAGCACAAAGGTG |
|  | R | TCCCAGCTTAACCTGTAGTCG |

Table S2. List of flow cytometry antibody details

| **Antibody** | **Fluorochrome** | **Clone** | **Host species** | **Source** |
| --- | --- | --- | --- | --- |
| CD45 | FITC | 30-F11 | Rat | BD Biosciences |
| CD11c | BV605 | HL3 | Hamster | BD Biosciences |
| CD11b | PE-Cy7 | M1/70 | Rat | BD Biosciences |
| Ly6G | PE | 1A8 | Rat | BD Biosciences |
| Ly6C | PerCP-Cy | AL-21 | Rat | BD Biosciences |
| F4/80 | BV421 | T45-2342 | Rat | BD Biosciences |
| CD64 | AF647 | X54-5/7.1 | Mouse | BD Biosciences |
| MHC II | BV510 | 2G9 | Rat | BD Biosciences |
| α7nAChR | AF647 | IgG_1_ | Rat | Santa Cruz  Biotechnology |
